# Diagnosis and Treatment of Pulmonary Tuberculosis

**Published:** 1906-07

**Authors:** H. F. Harris

**Affiliations:** Atlanta, Ga.


### DIAGNOSIS AND TREATMENT OF PULMONARY TUBERCULOSIS.

BY H. F. HARRIS, M.D.,
ATLANTA, GA.

The medical profession has been so often disappointed in the treatment of pulmonary tuberculosis with the ordinary remedies—has witnessed the rise, decline and fall of so many vaunted new specifics for this terrible disease, that it has reached a condition where failure is expected, and the whole subject of the therapy of this malady is regarded with apathetic indifference. To such an extent is this true that I find we have almost as a whole disregarded and overlooked the really wonderful advances in more recent times that have been made in the treatment of this affection.

The combat against this terrible foe that has placed us in a measurable degree of victory has not been carried on in the pseudo-scientific journals of "bio-chemistry," the morning gazettes of "the newer remedies," or the evening telegrams of the "alkaloidal specifics," nor yet in the daily lay newspapers, one of the special prerogatives which the vast majority of them exercise being the dissemination of misinformation generally, and more particularly on medical subjects. In our own land this seemingly hopeless combat was begun nearly three decades ago in the lonely and at that time uninhabited wilderness of the Adirondack mountains of northern New York, a combat to which no admiring onlookers lent applause, as silent and grim as the icy peaks of the surrounding hills. Here all alone, a weak and emaciated invalid, himself a sufferer from this frightful malady, struck the first blow in the great fight which has resulted in such unparalleled good to mankind, and which is destined in the future to be of infinitely more benefit to suffering humanity. All honor to Trudeau, one of the greatest philanthropists and physician of this or any other time. To him has fallen the happy lot of knowing that thousands are now strong and well who, but for him, would have long since passed away, the victims of one of the most terrible and loathsome of all diseases.

With this great example before us it is our duty to let the world know what can be done to combat the great scourge of tuberculosis, and it behooves us as true physicians to do all in our power to further the great ends that have been already so nobly striven for.

In order that this may be done, nothing is so essential as for us to inform ourselves as to all that is requisite in the recognition of tuberculosis in the earliest stages, and then impress upon our patients the necessity of prompt and energetic action. I think, then, that nothing could be more timely than for us to take up this subject, and with renewed ardor never allow it to rest for a moment so long as this great scourge continues its ravages among us.

Incipient tuberculosis is the form in which the disease is preeminently amenable to treatment, and it is, therefore, particularly to this period of the affection that our endeavors should be most directed in our attempts at its recognition. This incipient tuberculosis was defined at the last meeting of the National Association for the Study and Prevention of Tuberculosis, as follows:

"Slight initial lesion in the form of infiltration limited to the apex or a small part of one lobe.

"No tuberculous complications. Slight or no constitutional symptoms (particularly gastritic or intestinal disturbances or rapid loss of weight).

"Slight or no elevation of temperature or acceleration of pulse at any time during the twenty-four hours, especially after rest.

"Expectoration usually small in amount or absent.

"Tubercle bacilli may be present or absent."

*Diagnosis:* The symptoms observed at this period of the disease are usually so slight that they are altogether overlooked. The patient may, or may not, have observed that his general health is not quite up to the normal, or he may have noticed slight loss of appetite, or symptoms of indigestion; a cough is frequently complained of, and quite a number of patients expectorate blood-stained mucus; in some cases the pulse is slightly accelerated, and, particularly toward evening, the temperature may be somewhat elevated. The classical symptoms of the disease, such as loss of weight, anorexia, severe intestinal disturbances, cough, expectoration of quantities of thick, tenacious muco-purulent material, rapid respiration, fast pulse, sweats, fevers and chills come on during the latter stages of the affection.

In these incipient cases physical examination is of the utmost value in making a diagnosis. A careful examination of the apices of the lungs will generally reveal the presence of rales, which become particularly numerous after coughing. Over the infected region the respiration is usually somewhat prolonged, and of a higher pitch than normal, but in the very early stages of the affection these signs may not be observed. After a certain amount of consolidation has occurred more or less dullness may be made out. Typical bronchial breathing, or cavernous respiration, with flatness on percussion, are signs that will only be found in advanced cases.

Of course, the examination of sputum for the germ of tuberculosis is at all times of great value, and if the disease be suspected daily investigations should be made until the matter is definitely settled; in some cases it is only after a careful search of many weeks that the bacilli are finally found.

In the short period during which the world went wild on the subject of the treatment of consumption by tuberculin it was observed that among the most unpleasant effects produced by the injection of this substance were the rapid rise in temperature, fast pulse, severe pains in the muscles, and, in addition, increased number of rales in the chest. Similar effects were observed in tuberculous animals after injections of this substance; veterinarians quickly took advantage of this fact by injecting tuberculin for diagnostic purposes.

The results that followed the treatment of consumption with this agent were so disastrous that in the reaction that followed the drug was tabooed, and it is only quite recently that its value as a diagnostic agent in human tuberculosis is being generally recognized.

In using tuberculin for the purpose of diagnosis, the initial dose should be small—1 mg. being quite sufficient; if no symptoms follow 2 mg. may be administered after a period of two or three days, and if still no effects are produced 3, 5 and finally 10 mg. at intervals of several days may be given. Trudeau sometimes waits for several days and gives another dose of 10 mg. where the disease is strongly suspected, and no reaction has followed the previous injections.

When given cautiously in this way all experts on the subject agree that no ill effects follow, and as it offers the means of making a diagnosis before general symptoms, or physical signs make the nature of the malady clear, there can be no question of its great value, and that it should be generally employed for this purpose.

It is noteworthy that in more advanced cases the reaction fails to follow the administering of tuberculin, probably for the reason that the body is already so flooded with the poison of the germ that the addition of a small amount of tuberculin is not taken notice of by the system.

It affords me great pleasure to say that the Laboratory of the State Board of Health is now in process of manufacturing tuberculin, and that within a short time it will be supplied free to all physicians in Georgia.

I would like also to emphasize the fact, which our physicians do not seem to be generally aware of, that we are making free examinations at our laboratory for *tubercle bacilli,* and that reports are promptly furnished physicians who send sputum to us. Not only are these examinations of great importance to the unfortunate victim himself, but are of the utmost value to those who come in contact with him. I am sure that most of us have seen instances where an individual has contracted tuberculosis, and has undoubtedly infected other members of his family before the true nature of the malady was recognized; under such circumstances an early diagnosis with proper instructions to the patient as to general hygienic precautions and the disinfection of his sputum would undoubtedly prevent such disastrous occurrences.

*Treatment:* The treatment of tuberculosis along modern lines is marvelously simple; briefly stated, it consists in putting the patient practically out of doors night and day, in rest as long as the disease is active, and in supplying a sufficient quantity of good wholesome food. When treated in this way patients improve in an incredible degree in a very short period of time, and where the disease is truly incipient, six months to a year suffices for a complete cure in a large number of instances.

In a recent visit made to the great sanatoria of the North I was perfectly astounded to see the wonderful effects produced by this treatment. The patients appeared almost without exception perfectly well, and exhibited absolutely no outward evidence of the character of the disease from which they were suffering. I am sure it is not an overstatement when I assert that it would be almost impossible to collect together in any part of the world an equal number of individuals who would appear to be in such perfect health.

That we have in Georgia a large number of places where tuberculosis can be successfully treated there can be no question. The best informed all now agree that if the outdoor life, rest and proper feeding be carried out it makes comparatively little difference where the patient is treated; of course, it is admitted that good country air is best for tuberculous patients, as it is for every one else, but even this is not essential. For a majority of patients an altitude of from 1,500 to 2,500 feet is to be preferred, and fortunately we have a number of such places within our State which are easily accessible.

At the instance of Dr. L. G. Hardman, a member of this Association, the Legislature at its last session passed an Act ordering an investigation as to the feasibility of erecting a State Sanitorium for the treatment of tuberculosis, and I am quite sure that the bare statement of this fact will enlist the unanimous help of this Association in the support of this measure.

